# Patients' knowledge and perception on optic neuritis management before and after an information session

**DOI:** 10.1186/1471-2415-10-7

**Published:** 2010-03-21

**Authors:** Albert I Matti, Miriam C Keane, Helen McCarl, Pamela Klaer, Celia S Chen

**Affiliations:** 1 Department of Ophthalmology, Flinders Medical Centre, Flinders Drive, Bedford Park SA 5042, Australia; 2 NHMRC Centre of Clinical Eye Research, Flinders University, Bedford Park SA 5042, Australia; 3 The Multiple Sclerosis Society of South Australia and Northern Territory Inc 274 North East Road, Klemzig, SA 5087, Australia

## Abstract

**Background:**

Patients' understanding of their condition affect the choice of treatment. The aim of this study is to evaluate patients' understanding and treatment preferences before and after an information session on the treatment of acute optic neuritis.

**Methods:**

Participants were asked to complete a questionnaire consisting of 14 questions before and after an information session presented by a neuro-ophthalmologist. The information session highlighted the treatment options and the treatment effects based on the Optic Neuritis Treatment Trial in plain patient language. The information session stressed the finding that high dose intravenous steroid therapy accelerated visual recovery but does not change final vision and that treatment with oral prednisone alone resulted in a higher incidence of recurrent optic neuritis.

**Results:**

Before the information session, 23 (85%) participants knew that there was treatment available for ON and this increased to 27 (100%) after the information session. There were no significantly change in patients knowledge of symptoms of ON and purpose of treatment before and after the information session. Before the information session, 4 (14%) respondents reported they would like to be treated by oral steroid alone in the event of an optic neuritis and 5 (19%) did not respond. After the education session, only 1 patient (4%) indicated they would undergo treatment with oral steroid alone but 25 (92%) indicated they would undergo treatment with intravenous steroid treatment, alone or in combination with oral treatment. Results indicated that there were significant differences in the numbers of participants selecting that they would undergo treatment with a steroid injection (n = 22, p = 0.016).

**Conclusions:**

In this study, patients have shown good understanding of the symptoms and signs of optic neuritis. The finding that significant increases in the likelihood of patients engaging in best practice can be achieved with an information session is very important. This suggests that patient knowledge of available treatments and outcomes can play an important role in implementing and adopting guideline recommendations.

## Background

Integrating evidenced based practice into daily clinical care of patients is the driving force behind much needed clinical research. It is commonly assumed that clinical evidence ultimately transforms clinical management by health professionals and results in better patient outcomes. Recently it has been highlighted that evaluation of the impact of major clinical trials "Translational T2 Clinical Research" is essential when assessing the effects of interventions designed to improve quality of care [1,2]. Although such translational research is important to implement change, there are other barriers to change that may occur at the level of the patient, the clinician or the healthcare system [1,3].

Patient preferences in adopting the best practice for their own management is not known. Making evidence based decisions is a complex process integrating evidence based information with patient's circumstances and the individual patient's preferences [4-6]. Surveying patient understanding of their condition, their choice of treatment and barriers to treatment options is important to implement best practice.

Optic neuritis (ON) is an acute inflammation of the optic nerve resulting in painful loss of vision. Optic neuritis may be a clinically isolated syndrome but it can also be associated with Multiple Sclerosis (MS) which is a systemic demyelinating condition. In about a quarter of MS cases, the patient first presents with optic neuritis and over half of MS patients have at least one episode of optic neuritis during the course of their condition [7,8]. There is Level 1 evidence obtained from at least one properly designed randomized controlled trial [9] that treatment of an acute optic neuritis with intravenous corticosteroid followed by oral steroid results in faster vision recovery [10-12]. Optic neuritis is an ideal model to investigate patient understanding and belief as the signs and symptoms of optic neurits can be readily identified; the natural history of the condition and the effect of treatment with intravenous steroid have been studied [8,10,13]. Treatment with intravenous corticosteroid can help with faster vision recovery but does not change the final vision. As optic neuritis is a prominent feature of MS, which is a chronic condition that affects young people in the most productive years of their lives, and understanding patient preferences is essential to ensure good chronic disease management.

The aim of this study is to evaluate patient understanding and preferences regarding the treatment of acute optic neuritis and to determine whether a patient information session can assist in increasing the likelihood that patients will engage in guideline recommended best practice management.

## Methods

Patients newly enrolled with the Multiple Sclerosis Society of South Australia and Northern Territory from 1 November 2008 to 31 April 2009 were invited to a "Newly Diagnosed Multiple Sclerosis Information Evening". The society is a not for profit organization that provides a range of services including education and support for people who are newly diagnosed, and their families. The Newly Diagnosed Information Session is an education and support seminar which is organized twice a year and is tailored specifically to the person within 12 months of diagnosis of MS. The information session was developed in response to their clients' requests for information. It has been running for over 10 years. The current format of two invited guest speakers of the clients' choices (e.g. a neurologist, a physiotherapist, an ophthalmologist, a counsellor), followed by a panel discussion. During panel discussion time, participants can write down their questions or ask directly. This is facilitated by the senior MS Counsellor who has been running the session and found the process encourage participants to voice their questions.

Sixty newly diagnosed patients were registered with the organization and were invited to attend the information session. Prior to the session, participants were mailed a letter informing them of the study to understand participants' views on optic neuritis. Thirty people attended the session. At the start of the session, participants were invited to participate in a survey that comprised a questionnaire before and after an information session. Participation was entirely voluntary and this was clearly expressed to the participants on the front page of the Participant Information Sheet and Consent Form. The study was approved by the Flinders Clinical Research Ethics Committee.

Those who consented were given a survey of 14 questions on the diagnosis and treatment of acute optic neuritis (Additional File 1). The questionnaires were self-administered and for each question participants were given a list of responses to choose from. In several categories, respondents were allowed to select more than one response. Data was collected from each respondent, on the same variables before and after the information session. Data from before and after were linked by a unique identifying number on participant response forms. For each question, responses were tallied and presented as percentages of respondents who selected each option.

The information session consisted of a Powerpoint presentation presented by a neuro-ophthalmologist for 35 minutes. The content of the presentation included information about symptoms and signs of optic neuritis, and the treatment options available based on the Optic Neuritis Treatment Trial (ONTT) [14-16]. The pros and cons of different treatment options and the adverse effects of treatment were discussed. The natural history of optic neuritis recovery if the person did not receive any treatment were presented, outlining that non-treatment is an option. The information session highlighted that high dose intravenous steroid therapy accelerated visual recovery but did not change the long-term visual outcome. The findings that treatment with low dose oral prednisone alone had a significantly higher incidence of recurrent optic neuritis in the same or fellow eye were highlighted. There was a second Powerpoint presentation by a neurologist concentrating on immunomodulator therapy for 35 mintues. A short break of 20 mintues was taken for refreshments. During this time, a box is provided by the MS counsellor encouraging people to write down any questions they have. The presentation was followed by a panel discussion of 30 minutes with the neuro-ophthalmologist, the neurologist, an MS specialist nurse and counsellor, a physiotherapist, a social worker and an occupational therapist to answer any questions raised by the patients.

The guideline recommendation for treatment of acute optic neuritis includes no treatment or treatment with intravenous steroid injection [12]. Low dose oral treatment is not a guideline recommended management. The researchers predicted that attending an information session would increase the likelihood that a person with optic neuritis will be inclined to engage in guideline recommended treatment. In order to test this hypothesis, McNemar chi-squared tests were performed on before and after data relating specifically to these variables. Respondents with missing data were excluded from the corresponding analysis. Using Bonferroni's correction method to control for multiple tests, a p-value of less than 0.025 was considered significant.

## Results

A total of 27 participants completed the survey before and after the education session. The overall response rate was 90%. The majority of participants were aged between 31-60 years of age, with 11 (41%) of the study population in the 31 to 45 age group (Table 1).

**Table 1:** Participants' demographics.

Age:	Number (n = 27)	Percentage
15 - 30	5	18
31 - 45	11	41
46-60	10	37
>60	0	0
Missing	1	4

All participants were within 4-8 months of MS diagnosis. Of the participants surveyed, 19 (70%) had heard of the term optic neuritis before the information session and about half had optic neuritis in the past.

**Figure 1:**
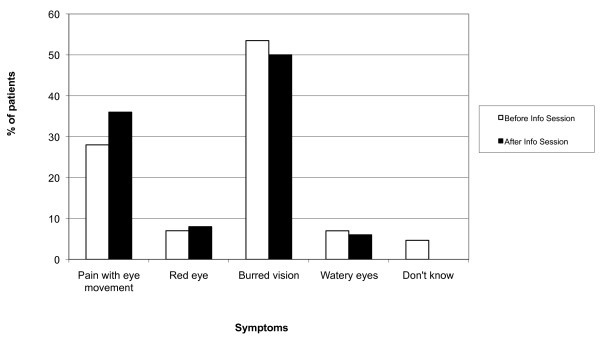
**Patient understanding of Optic Neuritis symptoms before and after the education session**. Figure shows percentage of participant responses to five ocular symptoms. There was no significant change in responses before and after the education session.

Participants had a good understanding of the symptoms associated with optic neuritis before the information session identifying blurred vision and pain with eye movement as the salient features of optic neuritis (Figure 1).

Table 2 shows participant understanding of various aspects of optic neuritis treatment before and after the information session. Before the information session, 23 (85%) participants knew that there was treatment available for ON and 2 people (7%) did not think there were treatments available. Two people (7%) did not respond. After the information session, all participants (100%) knew that there are treatments available.

**Table 2:** Participants' understanding of Optic Neuritis treatment before and after the Education session.

		Before (%)	After (%)
Do you think there is treatment available?			
	Yes	23 (86%)	27(100%)
	No	2 (7%)	0
	Non Responders	2 (7%)	0
Which option would you undergo? (choose one option)			
	Do nothing	3 (11%)	1 (4%)
	Oral steroid Tablets alone	4 (14%)	1 (4%)
	Steroid Injection	15 (56%)	25(92%)
	Non Responders	5 (19%)	0
When is treatment most effective from onset of symptoms? (choose one option)			
	Within 1 week	10 (37%)	19 (70%)
	Within 2 weeks	1 (4%)	1 (4%)
	Within 1 month	1 (4%)	(4%)
	Within 3 months	4 (14%)	2 (7%)
	Always effective	4 (15%)	4 (15%)
	Don't Know	5 (19%)	0
Non Responders	2 (7%)	0	
What are the purposes of treatment? (may choose more than one option)			
	Reduce further attacks	9 (33%)	8 (30%)
	Make vision recover to normal	7 (26%)	6 (22%)
	Make vision recover faster	17 (63%)	23 (85%)
	Pain relief	4 (15%)	3 (11%)
	Don't Know	5 (18%)	0
	Non Responders	1 (4%)	0

Participants were asked to choose which treatment options they would undergo in the event of an optic neuritis episode. Before the information session, 4 (14%) respondents reported they would elect to be treated by oral steroid alone in the event of an optic neuritis and 5 (19%) did not indicate how they would like to be treated. Fifteen (56%) indicated they would have intravenous treatment. After the education session, only 1 patient (4%) indicated they would undergo treatment with oral steroid alone. Twenty-five (92%) indicated they would undergo treatment with intravenous steroid treatment, either alone or in combination with oral treatment. One patient (4%) indicated they would do nothing in the event of acute optic neuritis. Results indicated that there were significant differences in the numbers of participants selecting that they would undergo treatment with a steroid injection (n = 22, p = 0.016).

Initially, only 10 (37%) participants believed that treatment is most effective if administered within 1 week of optic neuritis symptom onset. This increased to 19 (70%) after the information session. Results of the McNemar chi-square indicate that this was not significant (n = 25, p = 0.08).

In response to the purpose of treatment, most participants recognized that treatment makes vision recover faster and this was selected by 17 (63%) participants before the information session and increased to 23 (85%) after the information session. Before the information session, 5 (18%) respondents chose, "Don't know" as the response to purpose of treatment but this was reduced to zero after the information session. Of the choices given (Table 2), the choice, "Make vision recover to normal" is an incorrect belief. Before the information session, 7 (26%) chose the answer and 6 (22%) participants continue to believe so after the information session.

## Discussion

Increasing patient understanding of guideline recommended best practice is important in management of chronic medical conditions such as MS. This approach has the advantage of better patient compliance, greater satisfaction with the health care provider/patient interaction and this translates into an increase in the quality adjusted life years experienced by an MS sufferer. Awareness of the management options and rationales will result in increased adherence to guideline recommendations. Although there is some concern that providing evidenced based information to patients may increase their anxiety and distress, numerous studies in MS patients found no adverse emotional effects on patients [17,18]. It has also been shown that communicating with patients about medical evidence increases their knowledge regardless of the communication tools used [19].

In this study, more than half of the people with newly diagnosed MS had heard of the term optic neuritis before the information session and more than half reported previous optic neuritis. This highlights the high prevalence of optic neuritis as a presenting or relapsing feature in people with MS. Our study may have been biased in terms of the results of prior knowledge of optic neuritis given the high proportion of patients with previous optic neuritis. Hence there were no significant differences in symptom recognition after the information session. The treatment options emerged as key features from this survey that has important management implications. After the information session, more participants elect to be treated with intravenous corticosteroids as the preferred treatment option compared to before the information session. A significant number elect to be treated early after recognizing the symptoms of optic neuritis.

The information session highlighted the findings of the ONTT that showed oral corticosteroid increases the rate of relapse in the same or fellow eye by about 30%. Therefore, low dose oral steroid alone is not a recommended guideline management option. There was a reduction of patients who indicated they would want to be treated with oral steroid alone after the information session (14% to 4%). More importantly, all participants have chosen a management option after the information session (19% did not before the information session). Despite previous publications citing that treatment of oral steroid alone is not an evidenced based recommended practice, a survey of the preference of optic neuritis treatment amongst Australian and New Zealand ophthalmologists and neurologists, showed that about 20% of both ophthalmologists and neurologists were still prescribing low dose oral corticosteroids [3]. Similarly, in an international survey on the management of optic neuritis, low dose oral steroid of 1 mg/km/day was still the recommended treatment of up to 65% of neurologist and 45.5% of ophthalmologist [1]. There is a significant variability between countries and depending on if the physician is an ophthalmologist or a neurologist. Those who chose oral steroid alone as a treatment option before the information session may have received this as a prior treatment for their optic neuritis. Reassuringly, after the information session, less people indicate they would undergo the option of oral steroid alone. However, one participant continued to select this option and it is important to explore the potential reasons for this. Patients could have different views and interpretations of a treatment options. Appraisal of treatment effects may be counterbalanced by side effects and personal inconveniences governing the individual's final decision on treatment preference. If the reason for not taking up intravenous treatment is due to concern, such as a needle-phobia or fear of having to go into a hospital, then patient information regarding alternative options for "out of hospital" management such as a day suite or "Hospital at Home" services for the infusion, or reinforcement of the option of non-treatment, may help patients to adopt the best practice.

In this study, approximately a quarter of participants believed that treatment with corticosteroids improved their final visual outcome. This is in conflict with the ONTT findings presented which clearly emphasized that corticosteroids accelerate visual recovery but do not reduce long term recurrence. There is a significant step between information provided by the clinician and that interpreted by the patients. The information and patient education should not be offered solely on the basis of what professionals believe to be necessary for the patient to know, but also on the basis of what patients want to know. The patients' actual worries and uncertainties should be taken as a basis for providing information and answers. In optic neuritis, re-gaining vision is a genuine concern for the patient affected and hence may affect the person's adaptation of what they'd like to believe. In promoting patient self care management, the health care professional has an explicit educational role, as well as a monitoring role [20] to ensure not just information is provided, but that information is correctly interpreted. Trobe et *al *showed that 65% of neurologists and 45% of ophthalmologists also believed that steroids improve final visual outcome [21]. It is therefore not uncommon for both patients and clinicians to hold views different to those supported by the evidence from major clinical trials. In implementation of best evidenced-based practice, awareness and agreement need to come about before adoption and adherence [22].

This study highlights that patients' preferences play an integral role in their management. Chong et al. noted that current high quality clinical practice guidelines have not given sufficient weighting to patient preferences [23] and this may have accounted for barriers to implementing evidence based recommendations in clinical settings [24]. Chong et al. identified several barriers in integrating patient preferences into clinical guidelines. A key enabler in guideline uptake will be to recognize and include preference evidences. Omitting patients' preference excludes an important element of what constitutes "best practice" to a patient.

Patient information is an integral part of best practice. Ennis et al. noted that in chronic disease such as MS, patients are not scared by information about their condition but in contrast, are able to digest complex medical information [25]. Having the information accessible to the patient with relevant information programs catering for the patient is therefore an important step forward to implementing best guideline recommended practices.

The study has several limitations including the self reporting bias introduced with survey methodology. The target population of this survey were people with newly diagnosed MS; their responses were likely influenced by their first clinical experience and the treatment given. Therefore, the results may be less applicable to more experienced MS patients. Another possible limitation of the study is that only the most highly motivated patients attended the session hence raising the question of the generalizability of the study. In our study, the majority of the attendees have a good understanding of the symptoms of optic neuritis prior to the information session and hence no significant change in knowledge of the disease is seen before and after the information session. However, this remains an important issue even if it only concerns newly diagnosed highly motivated patients. A further limitation of the study is that the information session did not address uncertainties associated with the ONTT which may influence patients' preferences to treatment options.

## Conclusions

In this study, patients have shown good understanding of the symptoms and signs of optic neuritis. The finding that significant increases in the likelihood of patients engaging in best practice in their management can be achieved with a simple information session. This is important as patient knowledge of available treatment options and treatment effects can play an integral role to help adopting guideline recommendations in their management.

## Competing interests

The authors declare that they have no competing interests.

## Authors' contributions

AM carried out data acquisition, analysis, interpretation and drafted the manuscript. HM and PK organized the patient information session and help conduct the questionnaire. MK carried out the statistical analysis and the drafting of the manuscript. CC conceived of the study and participated in its design, coordination and helped to draft the manuscript. All authors read and approved the final manuscript.

## Pre-publication history

The pre-publication history for this paper can be accessed here:

http://www.biomedcentral.com/1471-2415/10/7/prepub

## Supplementary Material

Additional file 1 **Optic Neuritis Questionnaire administered before and after the information session**. The 14 item self-administered questionnaire before and after the information session on symptoms and managements of optic neuritis. Click here for file
