# Supplementary material for: Patients' knowledge and perception on optic neuritis management before and after an information session
Source: BMC Ophthalmol. 2010 Mar 21;10:7. doi: 10.1186/1471-2415-10-7 (PMC2854102; doi:10.1186/1471-2415-10-7)
Supplement: Additional file 1 — Optic Neuritis Questionnaire administered before and after the information session. The 14 item self-administered questionnaire before and after the information session on symptoms and managements of optic neuritis. [file 1471-2415-10-7-S1.DOC]

**PRE INFORMATION SESSION QUESTIONNAIRE**

Your age : 15-30 31-45 46-60 >60

Have you heard of the term optic neuritis? Yes No

Have you had optic neuritis before? Yes No

Which of the following symptom do you think could be optic neuritis? (You may choose more than one)

 Watery eyes Eye pain with eye movement  Red eyes

 Blurred vision  Watery eyes

Which health professional would you first contact if you think you have optic neuritis? (Choose one)

 My MS nurse  My neurologist

 My GP  My eye specialist

 My optometrist  No-one

When would you contact this person? (Choose one)

 As soon as possible  Within 1 week if symptom persists

 Within 1 month  Within 3 months

Do you think there is treatment available if you have optic neuritis?Yes No

If yes, what are the treatments options? (Choose more than one)

 Doing nothing / no treatment Oral steroid tablets Steroid injection

Which of the above option would you undergo if you have optic neuritis? (Choose one)

 Doing nothing / no treatment Oral steroid tablets Steroid injection

When do you think treatment would be most effective? (Choose one)

 Within 1 week of symptom  Within 2 weeks of symptoms

 Within 1 months of symptoms  Within 3 months of symptoms

 Treatment will always be effective  Don’t know

What are the purposes of treatment? (you may choose more than one)

 Reducing further optic neuritis attacks  Make the vision recover to normal

 Make the vision recover faster  Make the pain better

 Don’t know

# POST INFORMATION SESSION QUESTIONNAIRE

Which of the following symptom do you think could be optic neuritis?

 Watery eyes Eye pain with eye movement  Red eyes

 Blurred vision  Watery eyes

Which health professional would you first contact if you think you have optic neuritis?

 My MS nurse  My neurologist

 My GP  My eye specialist

 My optometrist  No-one

When would you contact this person?

 As soon as possible  Within 1 week if symptom persists

 Within 1 month  Within 3 months

Do you think there is treatment available if you have optic neuritis?Yes No

If yes, what are the treatments options?

 Doing nothing / no treatment Oral steroid tablets Steroid injection

Which of the above option would you undergo if you have optic neuritis?

 Doing nothing / no treatment Oral steroid tablets Steroid injection

When do you think treatment would be most effective? (choose one)

 Within 1 week of symptom  Within 2 weeks of symptoms

 Within 1 months of symptoms  Within 3 months of symptoms

 Treatment will always be effective  Don’t know

What are the purposes of treatment? (you may choose more than one)

 Reducing further optic neuritis attacks  Make the vision recover to normal

 Make the vision recover faster  Make the pain better

 Don’t know

What is your view about having an Action Plan in the event of a relapse?

 Not interested  Maybe useful but unsure

 Will consider  Will definitely have an action plan

If you are considering developing an action plan, who would you first contact if you have symptoms that you think may be due to an optic neuritis attack?

 My MS nurse  My neurologist

 My GP  My eye specialist

 My optometrist  No-one

Any comments : __________________________________________________________
